# 2D Halide Perovskites for High‐Performance Resistive Switching Memory and Artificial Synapse Applications

**DOI:** 10.1002/advs.202310263

**Published:** 2024-04-22

**Authors:** Bixin Li, Fei Xia, Bin Du, Shiyang Zhang, Lan Xu, Qiong Su, Dingke Zhang, Junliang Yang

**Affiliations:** ^1^ School of Physics and Chemistry Hunan First Normal University Changsha 410205 China; ^2^ Shaanxi Institute of Flexible Electronics (SIFE) Northwestern Polytechnical University (NPU) Xi'an Shaanxi 710072 China; ^3^ School of Physics Central South University 932 South Lushan Road Changsha Hunan 410083 China; ^4^ School of Materials Science and Engineering Xi'an Polytechnic University Xi'an 710048 China; ^5^ School of Physics and Electronic Engineering Chongqing Normal University Chongqing 401331 China

**Keywords:** 2D halide perovskite, artificial synapses, resistive switching memory, Ruddlesden–Popper

## Abstract

Metal halide perovskites (MHPs) are considered as promising candidates in the application of nonvolatile high‐density, low‐cost resistive switching (RS) memories and artificial synapses, resulting from their excellent electronic and optoelectronic properties including large light absorption coefficient, fast ion migration, long carrier diffusion length, low trap density, high defect tolerance. Among MHPs, 2D halide perovskites have exotic layered structure and great environment stability as compared with 3D counterparts. Herein, recent advances of 2D MHPs for the RS memories and artificial synapses realms are comprehensively summarized and discussed, as well as the layered structure properties and the related physical mechanisms are presented. Furthermore, the current issues and developing roadmap for the next‐generation 2D MHPs RS memories and artificial synapse are elucidated.

## Graphical abstract

2D metal halide perovskites are among the most rapidly emerging materials for resistive switching memory and artificial synapses applications. This timely review elucidates the recent progress of 2D metal halide perovskites in achieving high performance and enhanced stability in memory and synapses applications. The challenges and development directions are also discussed.

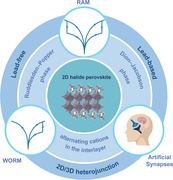

## 1 Introduction

Recently, the next generation of emerging nonvolatile memory technologies has been required to have large storage and high speed in order to meet the rapid development of big data, and artificial intelligence. Resistive switching memory (RSM) has been demonstrated to exhibit the advantages of simple structure, fast operation speed, low energy consumption, and long retention time.^[^ ^1^, ^2^, ^3^, ^4^, ^5^, ^6^ ^]^ Therefore, it has been extensively investigated for applications of nonvolatile memory with the ability to store and output information, as well as artificial synapses.^[^ ^7^, ^8^, ^9^, ^10^ ^]^ A representative RSM cell consists of an electrode/active layer/electrode sandwiched structure, which makes it easy to integrate into small passive crossbar arrays of 4*F* ^2^ (*F* is the minimum feature size).^[^ ^11^ ^]^ The RSM can switch reversibly between high‐resistance state (HRS, or “OFF” state) and low‐resistance state (LRS, or “ON” state) or among multiple resistance states under external electrical stimuli. According to the polarities of set and reset switching processes, random access memories (RAMs) can be divided into unipolar mode and bipolar mode. In unipolar memory, the electrical polarities during the set and reset processes should be same. Whereas the opposite electrical polarities lead to the bipolar memory.^[^ ^12^ ^]^

Tremendous materials have been used as the active layers in RSM, such as metal oxides, metal nitrides, transition metal chalcogenides, black phosphorus, organics, polymers, and metal halide perovskites (MHPs).^[^ ^13^, ^14^, ^15^, ^16^, ^17^, ^18^, ^19^, ^20^, ^21^, ^22^, ^23^, ^24^, ^25^, ^26^ ^]^ Among these materials, MHPs with the form of ABX_3_, where A is a monovalent cation, B is a bivalent cation, and X is a monovalent anion, have attracted extensive attention in the area of optoelectronic devices because of their excellent properties including long carrier diffusion lengths, tunable bandgap, balanced charge carrier mobility, strong absorption coefficient, and long carrier lifetime.^[^ ^27^, ^28^ ^]^ MHPs have been widely utilized in solar cells. The certified power conversion efficiency is exceeding 26%.^[^ ^29^ ^]^ Particularly, there is innate hysteresis inevitably in MHPs optoelectronic devices due to charge carrier traps, ion migration, or ferroelectricity. These properties render MHPs to be promising candidate for RSM application. Moreover, the facile fabrication process of MHPs on flexible substrates are core to construct the next‐generation wearable devices so as to integrate in our everyday outfits to achieve a higher comfort level.^[^ ^30^ ^]^

Recently, it has achieved promising resistive switching (RS) performance with ON/OFF ratio as high as 109 in 3D perovskites RSM.^[^ ^31^ ^]^ However, 3D perovskites suffer from the inherent limitations of poor reliability and stability in ambient atmosphere over moisture, light, and heat. The conducting filament pathway is hardly controlled in 3D perovskite, leading to poor endurance and retention.^[^ ^32^ ^]^ Accordingly, 2D perovskites with reduced dimensionality are effective to increase the stability of MHPs. By introducing large organic spacer cations into the A site of MHPs, they will form 2D layered structures with general formula of (RNH_3_)*_x_* A*_n_*_‐1_B*_n_* X_3_ *_n_*_+1_ (*n* = 1, 2, 3, …), where RNH_3_ is a large aliphatic or aromatic alkylammonium spacer cation (CH_3_(CH_2_)_n_NH_3_^+^, C_6_H_5_(CH_2_)_n_NH_3_^+^), A is a monovalent cation, B is a divalent metal cation, X is a halide anion, and *n* represents the number of octahedral layers within large organic spacer cations.^[^ ^33^ ^]^ Inserting hydrophobic organic spacer cations can hinder the permeating of oxygen and water. Thus, 2D MHPs generally exhibit much better moisture stability than their 3D counterparts. Besides, the charged defects are mostly distributed in the intralayer of the 2D layer structure. It could reduce the random migration of charged defects in 2D MHPs, which is profitable for the formation and rupture of a conducting filament.^[^ ^32^ ^]^ More importantly, because of the isolated nature of octahedral complexes and reduced trap densities, 2D MHPs often exhibit high intrinsic resistivity, which is benefit for facilely lowering the OFF current so as to increase the ON/OFF ratio in RSM. Therefore, 2D MHPs exhibit great potential in high‐performance RSM.

The typical hysteresis property in MHPs may enable gradual modulation of conductance. This characteristic encourages MHPs a promising candidate in neuromorphic computing. Neuromorphic computing systems that can mimic the human brain are considered to overcome the energy and throughput limitations of conventional serial von Neumann computing architecture. The human brain is a sophisticated neural network system with 1011 neurons and 1015 synapses.^[^ ^34^ ^]^ It can process huge amounts of information simultaneously, while consuming less than 20 W.^[^ ^35^ ^]^ Generally, there are different kinds of electronic devices configurations to realize the neuromorphic computing, such as magnetic memories, phase‐change, organic electrochemical devices, two‐terminal floating‐gate transistors, and memristors.^[^ ^36^, ^37^, ^38^, ^39^, ^40^ ^]^ Artificial synapses based on MHPs show synaptic responses with relatively low activation energy and low energy consumption of ≈20 fJ per synaptic event.^[^ ^41^ ^]^ By modulating the structure of MHPs, energy consumption could be further lowered to biological synaptic levels. Therefore, MHP synapses pave a plausible application for MHPs in neuromorphic devices.^[^ ^42^ ^]^

**Figure 1:**
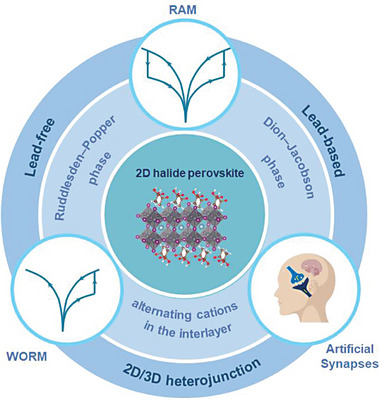
Overview of 2D MHPs memristors.

At present, there are some reviews focused on organic‐inorganic MHPs‐based RSM and artificial synapses,^[^ ^34^, ^43^, ^44^ ^]^ which are mainly concentrated on the advances of lead‐based and lead‐free MHPs, flexible perovskites memristors, and perovskites synaptic devices. Recently, organic‐inorganic 2D MHPs memristors and memristor‐based synaptic applications are being rapidly developed, and it is very necessary and meaningful to summarize their research progress and discuss the challenges and outlook. Herein, we comprehensively summarize the recent progress in RSMs and artificial synapses based on organic‐inorganic 2D MHPs, and the overview is depicted in **Figure** **1**. First, the molecular structures and the physical properties of 2D MHPs are illustrated. Then the important role of 2D MHPs in RSM and synaptic devices is reviewed with regard to material preparation, and device structure. Finally, the perspectives and outlooks of 2D MHPs based memories and artificial synapses are involved. It is believed that the insights will shed light on developing high‐performance memories and artificial synapses for the next‐generation electronics.

## 2 2D MHPs Structures and Properties

### 2.1 Layered Perovskite Structures

**Figure 2:**
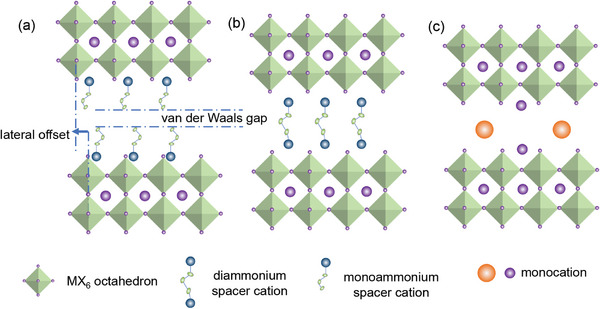
Illustration of 2D MHPs phases: a) RP phase; b) DJ phase; c) ACI phase.

The structure of 2D MHPs can be adjusted by designing the spacer cations molecular structure. It can be altered by changing the alkyl chain length, introducing ammonium dications or π‐conjugated segment. These properties greatly impact the electronic and optoelectronic properties of MHPs. Moreover, 2D MHPs can be classified into three types of structures including Ruddlesden–Popper (RP) phase, Dion–Jacobson (DJ) phase, and alternating cations in the interlayer (ACI), as shown in **Figure** **2**.^[^ ^45^, ^46^, ^47^ ^]^ Generally, they are composed of large cation layers and metal halide octahedral layers. The common monoammonium organic cations are *n*‐butylammonium (BA), iso‐butylammonium (iso‐BA), *n*‐octylammonium (OA), *n*‐hexylammonium (HA), phenylethylammonium (PEA), and phenylmethylammonium (PMA), which are presented as organic spacers in 2D RP phase perovskites. Similarly, (aminomethyl)piperidinium (AMP), (aminomethyl)pyridinium (AMPY), 1,4‐phenylenedimethanammonium (PDMA), and other short‐chain alkyldiammonium cations are used as organic spacers in 2D DJ layered perovskites.^[^ ^48^ ^]^

The RP phase is currently the most investigated 2D perovskite phase. The ammonium units combine with the inorganic layer through either ionic bonding or hydrogen bonding. Herein, van der Waals forces or π‐π interactions exist between the organic cation bilayer, resulting in some extent “frozen”. There is a lateral offset between the metal halide sheets by half an octahedral unit. Meanwhile, the van der Waals gap is formed between the opposite facing monoammonium cations within the organic bilayer.

In the DJ phase, there are hydrogen bonds formed between diammonium cations and the vicinal inorganic metal halide layers on both sides. Therefore, the DJ phase exhibits better structural stability as compared with RP phase. The single organic layer causes a partial or no lateral offset of the metal halide sheets. The interlayer distance of DJ phase perovskites is typically smaller and the structural distortion is lower associating with a lower bandgap than that of RP phase. However, because of the limited number of readily available diamines, the DJ phase has yet to be comprehensively studied.

The ACI phase constructed of two types of cations in the interlayer space with the formula of A'A*_n_* B*_n_* X_3_ *_n_*_+1_ has been investigated rather few. The A’‐site is usually the large cation such as guanidinium or 1,4‐butanediammonium.^[^ ^49^ ^]^ The A‐site is typically a smaller cation of methylammonium (MA) or formamidinium (FA).^[^ ^45^, ^50^, ^51^ ^]^ The interlayer distance is small in this phase with no lateral offset of metal halide octahedra layer.

### 2.2 Bandgap Engineering

**Figure 3:**
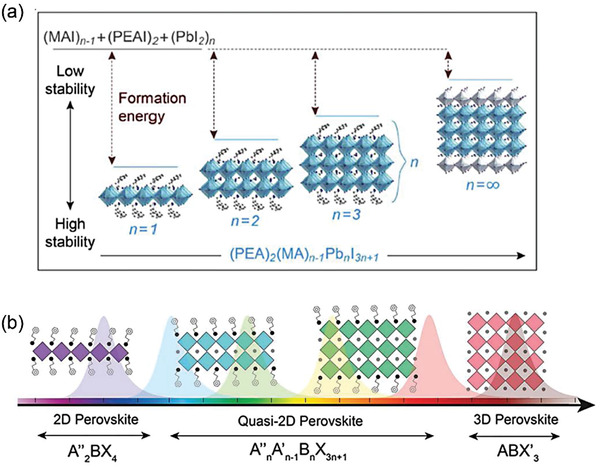
a) The evolution of dimensionality and formation energy as a function of *n*. Reprinted with permission.^[^ ^55^ ^]^ Copyright 2016, American Chemical Society. b) Schematic illustration of perovskites bandgap and dimensionality engineering relationship. Reprinted with permission.^[^ ^54^ ^]^ Copyright 2018, Wiley‐VCH.

Bandgap engineering for 2D MHPs plays an important role for electronic and optoelectronic applications. In 2D MHPs, the bandgap can be facilely adjusted by chemical composition, dimensionality, and nanostructure.^[^ ^52^ ^]^ Generally, changing A‐site cations leads to crystal structure transformation and tunable bandgap. By introducing the large spacer cation layers, the inorganic octahedral slabs would be separated, resulting in the multiple quantum wells structure.^[^ ^53^ ^]^ The inorganic octahedral slabs thickness (i.e., the *n* value in the chemical formula) and the dimensionality are affected by the ratio of small cations and spacer cations. With an increase of *n* from 1 to ∞, the RP phase perovskite transforms from 2D to a mixed 2D/3D or quasi‐2D and finally a 3D phase, the bandgap of perovskite decreases gradually, as shown in **Figure** **3**a,b.^[^ ^54^ ^]^ Take BA_2_MA*_n_*_−1_Pb*_n_* I_3_ *_n_*_+1_ perovskite for example, it is found that *E*g decreased with increasing *n* values, from 2.43 (*n* = 1) to 1.50 eV (*n* = ∞).^[^ ^33^ ^]^

Narrow bandgap perovskites are widely used in light‐emission devices, while wider bandgap perovskites are profitable in memory application. The large bandgap of 2D MHPs influences the Schottky barrier of the interface, the halide vacancies interaction energy and the conductivity. Generally, the ON/OFF ratio is an important parameter in memory device. With wider bandgap of 2D MHPs, a high Schottky barrier can be formed between the active layers and the electrodes, resulting in a feasible lower current at HRS.^[^ ^32^ ^]^ Therefore, the ON/OFF ratio would be elevated. We will discuss this property in detail in Section 4.1.1. Moreover, owing to the spatial isolation of octahedra, the transport of charge carriers is limited in the 2D plane. Therefore, the intrinsic charge conductivity toward out‐of‐plane direction of 2D perovskites are greatly suppressed, which is beneficial for low‐power consumption memory devices. More importantly, the halide vacancies are primary factors for halide vacancies‐based memory devices. The interaction energy between halide vacancies is lower in wider bandgap MHPs for halide vacancies in 2D perovskites can easily place within the bandgap. Finally, the lower interaction energy facilitates formation and breakage of halide vacancy conductive filaments (CFs), valuable for memory utilization.^[^ ^56^ ^]^

### 2.3 Enhanced Stability

Compared with conventional 3D MHPs, the most promising feature of 2D MHPs is their greatly enhanced stability, which is mainly attributed to the high formation energy and hydrophobic ammonium cations.^[^ ^57^ ^]^

The introduction of large organic spacer cations can successfully reduce the degradation of 2D perovskites by increasing the formation energy (Figure 3a). In the conventional 3D MHPs like MAPbI_3_, experimental and theoretical investigations have proved that the low formation energy resulting in the decomposition of their precursors MAI and PbI_2_.^[^ ^58^ ^]^ By introducing PEA into the perovskite layers, the mixed‐cationic perovskite material exhibits an increased formation energy to enhance the stability. A similar effect can also be observed by introducing BA.^[^ ^55^, ^59^ ^]^ Density functional theory calculations have shown that the perovskite film lifetime and formation energy obey the following laws.

(1)

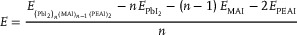




Therefore, MAI is much more easily to run away from the perovskite surface than PEAI, with decomposition energy of 2.15 and 2.51 eV, respectively.

In addition, 2D MHPs also exhibit better stability against moisture due to the hydrophobic ammonium cations. PEAI, a typical hydrophobic spacer cation, could provide moisture resistance and prolong the RSM device stability under ambient conditions from 5 days for 3D CsPbI_3_ to more than two weeks for quasi‐2D (PEA)_2_Cs_3_Pb_4_Br_13_.^[^ ^60^ ^]^ The hydrophobic spacer cations not only enlarge the perovskite bandgap but also act as a protection layer against ambient moistures. Therefore, the atmosphere stability of 2D perovskites has been greatly elevated, which is valuable for high stability RSM devices.

### 2.4 Versatile Structure and Composition

The 2D MHPs are not restricted by Goldschmidt's tolerance factor, $t=\left(r_{A}+r_{X}\right)/\left[\sqrt{2}\left(r_{B}+r_{X}\right)\right]$, where *r*_A_, *r*_B_, and *r*_X_ are the effective radii of A^+^, B^2+^, and X^−^ ions, respectively. However, this factor is obeyed in 3D MHPs.^[^ ^61^ ^]^ Herein, 2D MHPs can accommodate with both the organic and inorganic molecules as A‐site cations. The structure and composition are much richer in 2D MHPs family. The inserted functional molecules enrich the physical and electrical properties of 2D MHPs, leading to fruitful RSM phenomena.^[^ ^62^, ^63^ ^]^

## 3 Mechanisms of RSMs

The mechanisms of halide perovskite RSMs are complicated. Many mechanism models have been proposed. Among these mechanisms, they can be divided into conductive filamentary‐type switching and interface‐type switching. The mechanisms of the 2D MHPs RSMs are listed in **Table** **1**.

**Table 1:** Summary of memory parameters based on 2D halide perovskites.

Structure	Set/reset voltage [V]	ON/OFF ratio	Endurance (times)	Retention [s]	Mechanism	Device type	References	
FTO/ZnO/BA_2_PbBr_4_/NiO	1.2/−2	≈10	–	3 × 103	V_Br_ CF	WMRM	[67]	RP phase
ITO/BA_2_PbBr_4_/Au		2400	60	103	V_Br_ CF	WMRM	[66]	
Pt/BA_2_PbBr_4_/Ag‐doped ZnO/Al	0.3/−0.25	106	3 × 104	5 × 104	Ag CF	WMRM	[68]	
ITO/(RNH_3_)_2_(FA)_1_Pb_2_Br_7_/Au	1.38/–	104	–	–	V_Br_ CF	WORM	[86]	
Au/(PEA)_2_PbBr_4_(single crystal)/Graphene	2.8/–	102	100	103	V_Br_ CF	WMRM	[70]	
FTO/(PEA)_2_PbI_4_ (single crystal)/Au	4.2/−4.2	103	30	103	V_I_ CF	WMRM	[74]	
Si/SiO_2_/Ti/Pt/(BzA)_2_CuBr_4_/PMMA/Ag	0.21/−0.27	>108	2000	103	V_I_ CF	WMRM	[76]	
ITO/PEDOT:PSS/PEA_2_MA_4_Pb_5_I_16_/PCBM/Ag	0.2/−1	104	500	9 × 105	Ag CF	WMRM	[92]	
ITO/PEDOT:PSS/PEA_2_MA_11_Pb_10_Br_33_/Bphen/Ag	2/–	64	–	60	Ag CF	WMRM	[71]	
ITO/PEDOT:PSS/(PEA)_2_MA_4_Pb_5_I_16_/PCBM/Ag	0.4/−0.4	104	–	–	Ag CF	WMRM	[75]	
ITO/PEDOT:PSS/BA_2_MA_4_(Pb_0.5_Sn_0.5_)_5_I_16_/PMMA/Au	1.35/–	102	2000	105	Interface type	WMRM	[81]	
Si/SiO_2_/Ti/Pt/BA_2_CsAgBiBr_7_/Ag	0.13/−0.2	107	1000	2 × 104	Interface type	WMRM	[77]	
Si/SiO_2_/Ti/Pt/BA_2_PbI_4_/Ag	0.7/−0.8	107	250	103	Interface type	WMRM	[32]	
Si/Pt/(An)_2_PbI_4_/PMMA/Ag	0.3/−0.25	106	130	2 × 103	Ag CF	WMRM	[72]	
Si/Pt/(BzA)_2_PbI_4_/PMMA/Ag	0.2/−0.29	107	140	5 × 103	Ag CF	WMRM	[72]	
Si/Pt/(PEA)_2_PbI_4_/PMMA/Ag	0.2/−1	108	220	5.5 × 103	Ag CF	WMRM	[72]	
Si/SiO_2_/Ti/Pt/(PEA)_2_Cs_3_Pb_4_I_13_/Ag	0.18/−0.1	109	230	2 × 103	Interface type	WMRM	[60]	
ITO/MA_2_PbI_2_(SCN)_2_/Al	‐1.59, −3.2/–	10,3 107	108	104	Pb CF	WMRM	[87]	
ITO/(3AMP)PbI_4_/Al	0.5/−0.2	103	1000	103	V_I_ CF	WMRM	[88]	DJ phase
Si/SiO_2_/Ti/Pt/MAPbI_3_/(PEA)_2_PbI_4_/Ag	0.18/−0.11	106	2700	104	Ag CF	WMRM	[64]	2D/3D
ITO/MAPbI_3−x_Cl_x_/BAI/Al	0.79/−0.77	>103	300	104	Interface type	WMRM	[89]	

WMRM: write‐many times‐read‐many times.

There is a CF formed between top and bottom electrodes. The formation and rupture of the CF is responsible for the set and reset process in the memory device. The CFs can be formed by electrochemical metallization and the redox reactions. When the electrochemical active metals like Ag and Cu used in electrodes, they are ease to be oxidized to cations and then migrate toward the opposite electrode under the electric field.^[^ ^64^ ^]^ They will be reduced to metal atoms at the opposite electrode and then the metal filaments achieve. The switch from HRS to LRS occurs. With a reverse voltage, the CFs will decompose to recover their initial HRS state.

The CFs can also be formed by halide vacancies when the electrodes of the device are inert metals.^[^ ^65^ ^]^ With the voltage applied, the positively charged halide vacancies will gather near the negatively biased electrode. Therefore, there are halide‐deficient and halide‐rich regions in the device. When the halide vacancies continuously grow, the CFs form and set the device from to ON state. With the negative bias, the halide vacancies will migrate to the opposite direction and annihilate with the halide ions, leading to the rupture of the CF.

The interface‐type switching is influenced by the interface between the perovskite active layer and the electrode.^[^ ^63^ ^]^ The Schottky barrier of the interface plays a vital role. The Schottky barrier depends on the difference between the perovskite's electron affinity and the work function of metal electrodes. Gap states, and interface passivation can adjust the Schottky barrier height as well as contact resistance. Then, the transition between the HRS and LRS states occurs. The resistance of interface‐type RSMs shows typically electrode area dependent.

## 4 2D MHPs RSMs

Since the first 2D RP MHP BA_2_MA*_n_*_−1_Pb*_n_* I_3_ *_n_*_+1_ RSM reported in 2017, a wide range of 2D MHP materials have been used as the functional layers in RSMs, including RP phase, DJ phase, 2D/3D heterojunction, etc.^[^ ^32^ ^]^ In order to obtain high‐performance RSMs, various strategies have been utilized, such as perovskite composition engineering, interface engineering, and electrode optimization. Up to now, great achievements have been made. In this section, recent progress in 2D MHPs for memory applications will be comprehensively discussed, and the detailed memory parameters of 2D halide perovskites are summarized in Table 1.

### 4.1 2D RP MHPs RSMs

#### 4.1.1 BA Spacer Cation

2D RP MHPs gain an intensive interest in memory applications. The two representative organic spacer cations, *i.e*., BA and PEA, have been widely utilized in 2D MHPs RRAMs.

**Figure 4:**
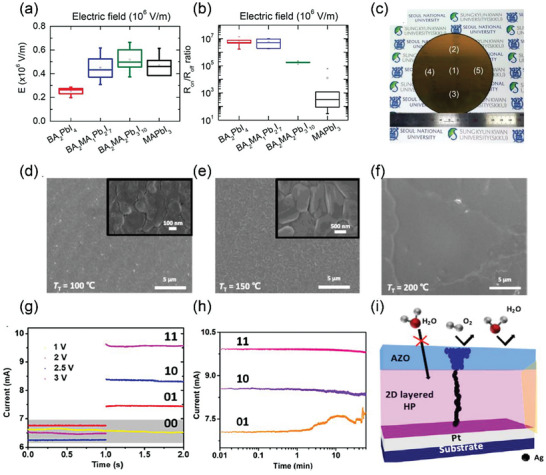
a) The electric field (*E*), and b) the ON/OFF ratio (under 0.02 V) for the different MHPs from BA_2_PbI_4_ to MAPbI_3_. c) 4 inch‐wafer BA_2_PbI_4_/Ag deposited on the (1) to (5). Reprinted with permission.^[^ ^32^ ^]^ Copyright 2017, The Royal Society of Chemistry. d–f) SEM images of the BA_2_PbBr_4_ film at 100, 150, and 200 °C, respectively. Reprinted with permission.^[^ ^66^ ^]^ Copyright 2019, American Chemical Society. g) Four different current states at different voltage. h) Retention stability of “11”, “10”, “01”. Reprinted with permission.^[^ ^67^ ^]^ Copyright 2019, American Chemical Society. i) Device structure of the switching device composed of BA_2_PbBr_4_ and the Ag‐doped ZnO (AZO) layer. Reprinted with permission.^[^ ^68^ ^]^ Copyright 2021, American Chemical Society.

Seo et al. changed the composition of BA_2_MA*_n_*_−1_Pb*_n_* I_3_ *_n_*_+1_ from *n* = 1 to *n* = ∞ to adjust the dimensionality and acquired the dimensionality‐dependent switching features.^[^ ^32^ ^]^ It was found that the 2D BA_2_PbI_4_ showed a more reliable and stable RS performance than 3D MAPbI_3_, with the lowest transition electric field of 2.5 × 105 V m^−1^, the highest ON/OFF ratio of 10,7 reliable endurance of 250 cycles, and retention of more than 103 s, as shown in **Figure** **4**a,b. Upon reducing the dimensionality from 3D to 2D, the bandgap of MHPs gradually increases. Therefore, the Schottky barrier heights between the MHPs and electrodes increase, leading to lower the HRS current. Meanwhile, the temperature‐dependent electrical measurements convinced the highest thermal activation energy in 2D MHPs. The lower Schottky barrier heights and higher thermal activation energy are responsible for achieving the high ON/OFF ratio. Interestingly, BA_2_PbI_4_ also exhibits reliable RS from 4 inch wafer‐scale under both room temperature and a high temperature of 87 °C (Figure 4c), which strongly indicates that 2D BA_2_PbI_4_ is a promising candidate for low‐cost and large‐area RSM.

The effects of the grain boundary and the grain size of 2D BA_2_PbBr_4_ on the RS behaviors have been investigated by Lee et al.^[^ ^66^ ^]^ The active perovskite film was deposited by using sequential vapor deposition process. The PbBr_2_ film is first deposited, followed by *n*‐butylammonium bromide (BABr) vapor‐assisted transformation in a glove box. The grain sizes could be well controlled by setting the temperatures at 100, 150, and 200 °C, achieving the BA_2_PbBr_4_ grain sizes of ≈180 nm, ≈1, and ≈30 µm, respectively (Figure 4d,f). With increasing the grain size, the OFF current decreases from ≈10^−4^ to ≈10^−8^ A. Therefore, the ON/OFF ratio of the RS devices is elevated by four orders of magnitude (Figure 4f). The migration of halide vacancies is attributed to the formation and rupture of conducting filaments in the RS devices. With a larger grain size, the number of grain boundaries decreases, leading to a decreased number of CFs and herein a lower OFF current.

By changing the electrodes and interface engineering, the devices based on 2D BA_2_PbBr_4_ with the structure of FTO/ZnO/BA_2_PbBr_4_/NiO exhibit a compliance‐free multileveled RS property.^[^ ^67^ ^]^ The device's average transmittance is over 70% across the visible range. The device shows set voltage of 1.2 V, reset voltage of ‐2 V, and retention time of 3000 s. Depending on the magnitude of applied voltage pulse, the device exhibits four different current levels as “00”, “01”, “10”, and “11”, which remains stable for a long time over 3000 s (Figure 4g,h), indicating multilevel RS. Kumar et al. ascribed the reason to the Br ion migration CF formation. The four different current levels are caused by the formation of different filament sizes, which is caused by the increasing ionic migration under relatively higher voltage. The similar RS characteristic can also be performed in a transparent multilevel ultra‐violet perovskite photodetector with the same device structure.^[^ ^69^ ^]^

By controlling CF formation in the active BA_2_PbBr_4_ layer, it opens a new avenue for improving the endurance and stability of the device. Figure 4i shows a device with the structure of Pt/BA_2_PbBr_4_/AZO/Al, and the AZO layer exhibits as a Ag ion reservoir to control the CF formation in the active layer by tuning the concentration of Ag in AZO.^[^ ^68^ ^]^ Moreover, it acts as a passivation layer on top of the active layer and protects the BA_2_PbBr_4_ layer from the ambient air. Therefore, the device exhibits a stable RS behavior over 3 × 104 cycles and shows no degradation for 15 days, which is much superior than the device with only an Ag electrode and without the AZO layer lasting only 4 × 102 cycles. A thick and stable filament in the active layer is responsible for resistive switching, whereas a thin and unstable filament is in charge of threshold switching. These behaviors could be realized by modifying concentration of Ag in AZO during the cosputtering process.

#### 4.1.2 PEA Spacer Cation

**Figure 5:**
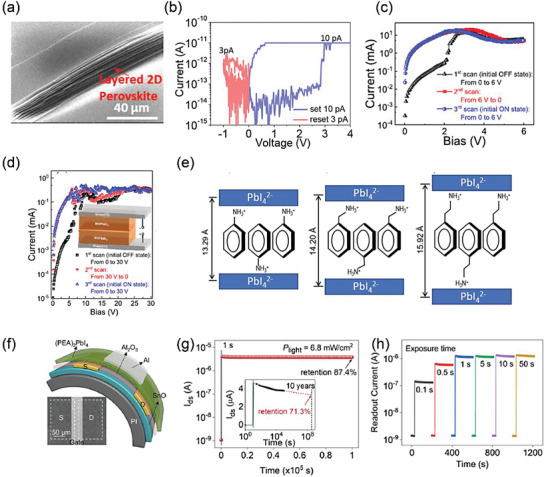
a) SEM image of an PEA_2_PbBr_4_ single crystal. b) Typical electrical curves for set and reset process of PEA_2_PbBr_4_ single crystal device. Reprinted with permission.^[^ ^70^ ^]^ Copyright 2017, American Chemical Society. c) *I−V* curves under three different scans: from 0 to 6 V with an initial OFF state and from 6 to 0 V and from 0 to 6 V with an initial ON state for ITO/PEDOT:PSS/perovskite/Bphen/Ag device. d) *I−V* curves under three different scans. The inset shows the device structure of ITO/MAPbBr_3_/MAPbBr_3_/ITO. Reprinted with permission.^[^ ^71^ ^]^ Copyright 2020, American Chemical Society. e) Schematic illustration of the interlayer structure of (An)_2_PbI_4_, (BzA)_2_PbI_4_, and (PEA)_2_PbI_4_. Reprinted with permission.^[^ ^72^ ^]^ Copyright 2019, The Royal Society of Chemistry. f) Device structure and SEM image of flexible memory. g) The storage stability of the memory behavior (P_light_ = 6.8 mW cm^−2^). h) Multilevel memory property of the optoelectronic memory. Reprinted with permission.^[^ ^73^ ^]^ Copyright 2022, American Chemical Society.

Apart from BA, PEA spacer cation in 2D MHPs is gained increasing interest in RS devices. In 2017, Tian et al. first investigated an extremely low operating current RS based on exfoliated 2D PEA_2_PbBr_4_ single crystals with the structure of Au/PEA_2_PbBr_4_/Graphene.^[^ ^70^ ^]^ Toward the out‐of‐plane direction of the 2D perovskite film, the charge transport is suppressed. Hence the OFF current is lowered, which enables the RSM to work at 10 pA, an extremely low currents with a switching ratio of 10, as shown in **Figure** **5**a,b. In the recent reported 2D perovskite RSMs, the 10 pA operation current is the lowes. The Br ion migration leads to the formation of Br vacancy CFs, which could be verified by Monte Carlo simulations and the cross‐sectional scanning transmission electron microscopy (SEM) with a diameter of 20 nm.

The PEA_2_PbI_4_ single crystals also shows reliable ON/OFF ratio of 103 with FTO/PEA_2_PbBr_4_/Au configuration.^[^ ^74^ ^]^ Replacing FTO with Au to make a symmetric device configuration, the Au/(PEA)_2_PbI_4_/Au device exhibits asymmetric bipolar RS curves. The CFs formed by I anion migration as well as charge trapping are responsible for the memory phenomenon. Under external electric field, I^−^ ions migrates and I^−^ vacancies in the inorganic layer forms conducting filament. This device can be set to various current levels, leading to the possibility to achieve multilevel storage.

Solanki et al. explored the RS feature in PEA_2_MA*_n_*_−1_Pb*_n_* I_3_ *_n_*_+1_ 2D perovskites with an ITO/PEDOT:PSS/perovskite/PCBM/Ag device configuration.^[^ ^75^ ^]^ When *n *= 5, the sample shows the champion RS performance with an ON/OFF ratio of ≈104 under a 0.15 V set voltage, endurance cycles of 500, and retention time over 105 s for 50 h under 60% relative humidity. In 2D RP MHP films, the conducting layer, nonconducting layer, and their alignment decide the bulk conductivity. They performed the impedance spectroscopy to reveal that the interface barrier to control the resistive states. The barrier was decided by the chemical reaction between ions and the eletrodes. Replacing the top electrode as Au or a much thicker PCBM, no switching phenomenon could be found in the device. Therefore, they provided a design criterion in choosing materials to build up high‐performance RRAMs.

The perovskite film usually shows bipolar switching feature, whereas the unipolar switching can be hardly seen in this film. Yu et al. found that unipolar switching behaviors was shown in the low conductivity of a polycrystalline 2D perovskite layer.^[^ ^71^ ^]^ Insulating long‐chain ligands PEABr could block carrier transport in quasi‐2D PEA_2_MA_11_Pb_10_Br_33_the perovskite film. Interestingly, ITO/PEDOT:PSS/perovskite/Bphen(20 nm)/Ag with stable unipolar RS property is realized in the device with 2 V set voltage, switching ratio of 64, and retention time of 60 s, as shown in Figure 5c. Moreover, they also fabricated two identical structures of ITO/MAPbBr_3_ and combined two MAPbBr_3_ layers with an architecture of ITO/MAPbBr_3_/MAPbBr_3_/ITO. Similarly, Figure 5d shows that the symmetrical device exhibited excellent unipolar switching behaviors with ≈7.5 V threshold voltage. Under high external bias, various 100 nm small grains were formed from 10 µm large perovskite grains to behave as microconducting channels, which is responsible for unipolar RS behavior. Both the bulk and the microchannels contribute to the current. In the low conductivity region, the current in the microchannels will be enhanced and brings large Joule heating. Thus, the same sweeping directional bias can turn off the device in HRS at high voltages, leading to unipolar switching.

Most perovskite‐based devices are reported with ON/OFF ratios between 104 and 10,7 which are still too low to commercialize. Jang's group compared the RS devices based on quasi‐2D perovskite PEA_2_Cs_3_Pb_4_I_13_ and 3D CsPbI_3_, and it was found that the PEA_2_Cs_3_Pb_4_I_13_ showed the highest ON/OFF ratio of 10,9 much higher than that of CsPbI_3_.^[^ ^60^ ^]^ In quasi‐2D, the Schottky barrier height increases at the interface between the active layer and Ag electrode, rendering a decreased HRS current. As a result, the quasi‐2D shows high ON/OFF ratio. Additionally, PEA_2_Cs_3_Pb_4_I_13_ also exhibits long‐term stability for 2 weeks under ambient conditions, superior than that of CsPbI_3_ with only 5 days.

The dimensionality on the RS properties have been thoroughly investigated. Meanwhile, it is also very meaningful to study the effect of the interlayer spacing in 2D perovskites influenced by the alkyl chain length. Kim et al. reported the dependence of RS on the alkyl chain length of alkyl benzene in [C_6_H_5_(CH_2_)*_n_* NH_3_]_2_PbI_4_ (*n* = 0, 1 and 2), where *n* = 0 for anilinium (An), *n* = 1 for benzylammonium (BzA), and *n* = 2 for PEA.^[^ ^72^ ^]^ It can be seen from Figure 5e that X‐ray diffraction measurements revealed that the interlayer spacing (*d*‐spacing) of inorganic (PbI_4_^2−^) sheets are 13.29 Å for An, 14.20 Å for BzA, and 15.92 Å for PEA, respectively. Interestingly, with increasing the interlayer spacing, the ON/OFF ratio is increased from 106 to 108 due to the gradual decrease of current of the HRS. The large interlayer spacing is beneficial for enhanced endurance and retention. The Ag CF is assumed to dominate the switching phenomenon.

Besides active layer, 2D RP MHPs can be utilized as the dielectric layer in ambipolar transistor‐based nonvolatile memories as well. The first typical work was conducted by Tian et al. with an Al_2_O_3_/PEA_2_PbI_4_ heterostructure dielectric configuration in a transistor‐type optoelectronic memory (Figure 5f).^[^ ^73^ ^]^ Magnetron sputtered bipolar SnO film was prepared as a conducting channel in the transistor. The photoexcited ions and electron−hole pairs will separate at the Al_2_O_3_/PEA_2_PbI_4_ interface. The accumulated carriers at the interface can gate the SnO channel and introduced a high specific detectivity of 2.7 × 1015 J. In the storage performance test of Figure 5g, the device was exposed to 532 nm laser pulse at *V*_gs_ = −5 V and *P*_light_ = 6.8 mW cm^−2^ for   s. When the laser is turned off, the *I*_ds_ is nearly in a steady state. In Figure 5h, the device also shows the multibit memory capacity under an ultralow power density of 25 µW cm^−2^, which means a superior optical storage ability. This work explores the perovskite‐based dielectric engineering for multilevel broadband‐response optoelectronic information memories.

#### 4.1.3 Lead‐Free 2D RP MHP RRAMs

**Figure 6:**
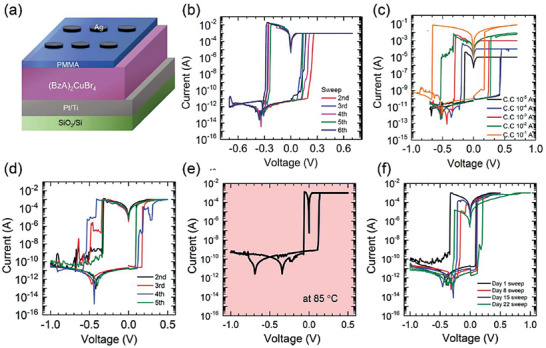
a) Schematic illustration of Ag/PMMA/(BzA)_2_CuBr_4_/Pt/Ti/SiO_2_/Si. b) *I–V* characteristics. c) *I–V* characteristics under different compliance currents. Reprinted with permission.^[^ ^76^ ^]^ Copyright 2020, WILEY‐VCH. d) *I–V* characteristics of Ag/BA_2_CsAgBiBr_7_/Pt. e,f) *I–V* characteristics of the device operated at 85 °C and for 22 days. Reprinted with permission.^[^ ^77^ ^]^ Copyright 2021, The Royal Society of Chemistry.

Although the 2D RP MHPs have shown outstanding RS properties, the toxicity of lead in MHPs is still a problem. No matter how small the amount of the lead, we cannot ignore potential harm to the environment. Therefore, some efforts on the lead‐free MHPs have been conducted. Generally, the lead (II) can be replaced by less‐ or non‐toxic elements such as tin (II), antimony (III), or bismuth (III).^[^ ^76^, ^77^, ^78^, ^79^ ^]^ But bismuth (III) and antimony (III) are hard to form 2D RP structure. Park's group found that copper (II) with 3d electronic configuration could form 2D layered perovskite.^[^ ^76^ ^]^ The octahedral Cu (II) is expected to present Jahn–Teller distortion.^[^ ^80^ ^]^ The device configuration of Si/SiO_2_/Ti/Pt/(BzA)_2_CuBr_4_/PMMA/Ag exhibits bipolar RS characteristics with 10^8^ ON/OFF ratio at millivolt scale, as shown in **Figure** **6**a,b. By changing the compliance current in Figure 6c, 5‐level storage characteristics could be achieved in the range of 10^−5^ to 10^−1^ A by 10^−1^ A interval to enhance the storage capability. The Ag CF f is responsible for the RS mechanism. (BzA)_2_CuBr_4_ shows superior ON/OFF ratio and endurance among various lead‐free perovskites, indicating that it is effective for environmentally friendly multilevel data storage RSM.

They also discovered RS property in lead‐free 2D double perovskite BA_2_CsAgBiBr_7_.^[^ ^77^ ^]^ In A_2_M(I)M(III)X_6_ double halide perovskite, two divalent Pb^2+^ cations are replaced by one monovalent and one trivalent cation. In order to reduce the dimensionality of Cs_2_AgBiBr_6_, they introduced BA into the 2D perovskite to form a double perovskite. The reduction of dimensionality enabled a high ON/OFF ratio of 10^7^ as comparable with that of (BzA)_2_CuBr_4_, as shown in Figure 6d. The HRS to LRS transition shows the Schottky conduction to Ohmic conduction, which is responsible for RS. The device is stable under 30–50% humidity for 22 days and at 85 °C (Figure 6e,f). However, the HRS exhibits large fluctuation at 10^8^–10^10^ Ω with incomplete rupture of conducting filaments at the reset process. Further enhancement of endurance is rather required.

By partly replacing Pb with Sn, Chen et al. studied the lead‐tin perovskite BA_2_MA_4_(Pb_0.5_Sn_0.5_)_5_I_16_ based memory property with the structure of ITO/PEDOT:PSS/BA_2_MA_4_(Pb_0.5_Sn_0.5_)_5_I_16_/PMMA/Au by innovative blade coating method.^[^ ^81^ ^]^ The insulating PMMA layer could lower the HRS current and elevate the ON/OFF ratio. By changing the ratio of Pb and Sn, they found that the pure Pb‐Sn perovskite shows superior RS properties than the pure Sn perovskite. They obtained a good RSM with set voltage of 1.35 V, 2000 cycles endurance, retention 105 s. Most importantly, the device shows good storage stability of 3 months in inert atmosphere. Log *I*–log *V* characteristics, impedance spectroscopy, photoluminescence spectra, and current response under laser light illumination are conducted to prove the defects in the perovskite layer. The injected carriers are trapped in the defects with increasing voltage. Upon the set voltage, all the traps are filled by charge carriers and a conversion from HRS to LRS occurs. The iodine ions migration also contributes in this process, causing a mix ionic and electronic conduction in the RSM behavior. The ion conduction process is certified by the impedance spectroscopy.

#### 4.1.4 Other Types of 2D RP MHPs

**Figure 7:**
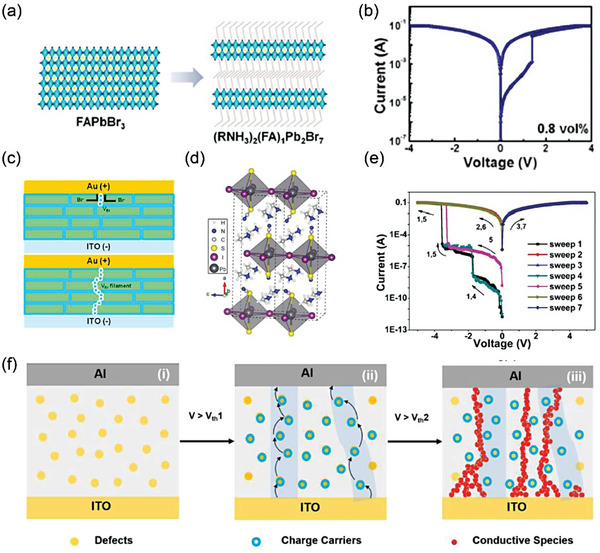
a) Schematic of FAPbBr_3_ and (RNH_3_)_2_(FA)_1_PbBr_7_ molecular structures. b) *I−V* curve of quasi‐2D (RNH_3_)_2_(FA)_1_Pb_2_Br_7_ with ligand concentrations of 0.8 vol %. c) Illustration of the formation of V_Br_ filaments. Reprinted with permission.^[^ ^86^ ^]^ Copyright 2021, American Chemical Society. d) Crystal structure of MA_2_PbI_2_(SCN)_2_ and orientation. e) Typical *I–V* curve of MA_2_PbI_2_(SCN)_2_‐based memory devices. f) Schematic diagram of MA_2_PbI_2_(SCN)_2_‐based memory mechanism. Reprinted with permission.^[^ ^87^ ^]^ Copyright 2018, Wiley‐VCH.

Different from introducing large spacer cations, ligands in 2D MHPs is an efficient way to improve RS memories performance.^[^ ^82^ ^]^ Halogen ions or organic materials and the Pb cations in perovskites could passivate the perovskite grains.^[^ ^83^, ^84^, ^85^ ^]^ In general, these ligands are larger than organic spacer cations and would compete with organic cations in crystal nucleation. Jeong et al. developed a novel method of a binary ligand solution for fabricating 2D perovskite films.^[^ ^86^ ^]^ They used the acid−base reaction of a binary ligand oleylamine and oleic acid solution in toluene to form oleylammonium (RNH_3_^+^) and finally a quasi‐2D (RNH_3_)_2_(FA)_1_Pb_2_Br_7_ was achieved, as shown in **Figure** **7**a. The structure of ITO/(RNH_3_)_2_(FA)_1_Pb_2_Br_7_/Au exhibits write‐once‐read‐many times (WORM) memory property (Figure 7b). The ON/OFF ratio gradually increased with the increasing ligand concentration and saturated at 10^3^ under 1.2 vol %. The low trap density in quasi‐2D perovskite is filled by injected charge carriers to form CFs, resulting in WORM phenomenon, as illustrated in Figure 7c.

Substituting halides in 3D perovskites with larger groups such as pseudohalide (SCN^−^) can also reduce them to 2D structure with improving film stability. 2D hybrid perovskites MA_2_PbI_2_(SCN)_2_ was fabricated, and promised as high effective device yield (75%) ternary memory, which is shown in Figure 7d,e.^[^ ^87^ ^]^ The crystal is grown layer‐by‐layer and each layer is composed of Pb(SCN)_2_I_4_ polyoctahedral sheet with MA^+^ separated, indicating the 2D layered structure. These layers mainly lied parallel to bottom ITO substrates. The organic cations hinder the out‐of‐plane direction charge transport, which is ease to form HRS. Thus, the device exhibits a current ratio of 1:103:107 in three different resistance levels of “0”, “1”, and “2”. It was found that the charge trap filling and the metallic filaments were responsible for this switch (Figure 7f).

### 4.2 2D DJ MHPs

**Figure 8:**
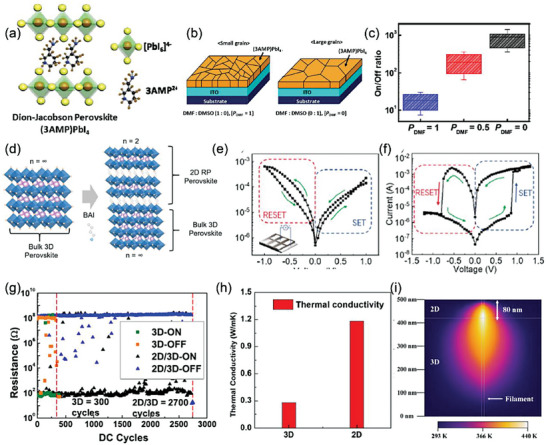
a) Schematic illustration of (3AMP)PbI_4_. b) Schematic demonstration of the grain size of (3AMP)PbI_4_ films under different volume ratios. c) Statistical distribution of ON/OFF ratio under different *V*_DMF_: *V*_DMSO_. Reprinted with permission.^[^ ^88^ ^]^ Copyright 2022, American Chemical Society. d) Schematic illustration of 2D RP perovskite formation process. e,f) *I−V* curves of devices fabricated by e) MAPbI_3−x_Cl_x_ and f) 2D/3D heterostructure structure. Reprinted with permission.^[^ ^89^ ^]^ Copyright 2020, American Chemical Society. g) Endurance behavior measured with the DC pulses for 2D and 2D/3D RS devices. h) Thermal conductivity of 2D and 3D perovskite films. i) Graph of the COMSOL simulation of the 2D/3D structure. Reprinted with permission.^[^ ^64^ ^]^ Copyright 2020, American Chemical Society.

Lee's group firstly investigated a DJ perovskite RS memories and revealed the grain size dependent RS features, just like the investigation on BA_2_PbBr_4_ showing the similar grain size dependence.^[^ ^88^ ^]^ An organic cation of 3‐(aminomethyl)piperidinium (3AMP) is introduced to fabricate (3AMP)PbI_4_ of DJ phase (**Figure** **8**a,b). In order to control the grain sizes, the *V*_DMF_: *V*_DMSO_ in the precursor are set as 1:0, 1:1, and 0:1. The grain sizes under the three volume ratios are 434 nm, 2.3 µm, and 13.3 µm, respectively. The ON/OFF ratio is also enhanced with increasing grain sizes (Figure 8c), as well as the promising endurance (1000 cycles) and data retention time (1000 s). The (3AMP)PbI_4_ also shows attractive properties in cross‐point memory array with 1S1R structure for high‐density memory applications.

The existed van der Waals gap can influence the stability of the 2D RP film. In contrast, the stability of DJ MHPs can be improved owing to the direct combination of adjacent inorganic perovskite.^[^ ^90^ ^]^ 2D DJ MHPs have been widely used in solar cells, while they are rarely applied in memories.^[^ ^91^ ^]^ Until now, only one work focused on 2D DJ MHP RS device was reported. Lee's work proved that the cycling endurance of the DJ MHP RS device could be enhanced compared with the RP MHPs counterparts. No degradation in RS behaviors in ambient air was observed even after 10 days. This result demonstrates the feasibility of using DJ MHPs in high‐performance memory applications.

### 4.3 2D/3D MHPs Heterojunction

In perovskite solar cells, the 2D/3D perovskite heterostructure can improve the stability and efficiency by the passivation effect. This heterostructure can also be introduced into RSM devices to improve the memory properties and fully utilize the superiorities of 3D and 2D perovskites. A 2D/3D heterostructure (BA)_2_MA*_n_*_−1_Pb*_n_* X_3_ *_n_*_−1_/CH_3_NH_3_PbI_3−_ *_x_* Cl*_x_* was fabricated by depositing *n*‐butylammonium iodide (BAI) on CH_3_NH_3_PbI_3−_ *_x_* Cl*_x_* film (Figure 8d).^[^ ^89^ ^]^ It can be clearly seen from Figure 8e,f that the ON/OFF ratio is increased by 2 orders of magnitude from 10 for pristine 3D perovskite to 103 for the 2D/3D heterostructure. The passivation effect could reduce the density of defects within the 3D layer. The HRS conduction switches from space charge limited conduction to Schottky emission transport. In the set process, the increasing voltage reduces the interface Schottky barrier height. Hence it induces charge tunneling and leads to LRS with Ohmic conduction. This work proved that the 2D/3D perovskite heterostructure passivation could improve the memory performance.

Besides ON/OFF ratio, the endurance and retention can also be enhanced by the 2D/3D perovskite heterostructure.^[^ ^64^ ^]^ A PEAI layer was fabricated on MAPbI_3_ film to form PEA*_n_*_+1_Pb*_n_* I_3_ *_n_*_+1_/MAPbI_3_ heterojunction. Dramatically, the ON/OFF ratio shows nearly no change, but the endurance is greatly enhanced from 300 cycles for the pure 3D device to more than 2700 cycles for the 2D/3D heterostructure as well as the improved retention and stability, as shown in Figure 8g. Compared with 3D perovskite, a higher hopping activation energy exhibits in 2D/3D heterostructure have and limits the Ag ion migration. Therefore, the Ag filament is narrow and the chemical reaction rate is rather slow between Ag and perovskite. As shown in Figure 8h, the thermal conductivity of 2D perovskite is 1.18 W m^−1^ K^−1^. Whereas, it is only 0.28 W m^−1^ K^−1^ for 3D perovskite. This difference results in a stronger Joule heat around the 2D perovskite film. The Ag filaments rupture in 2D films can be controlled better during the reset process. The COMSOL simulation further confirms this effect on the 2D/3D perovskite films. As shown in Figure 8i, the Joule heating speeds up the thermal dissolve of the Ag ions in the reset process. Therefore, it is beneficial for enhancing the endurance of MHP‐based RSMs.

## 5 2D MHPs Artificial Synapses

The hysteresis behavior of MHPs can be explored for application in neuromorphic electronics with a low operating voltage.^[^ ^93^, ^94^ ^]^ In order to mimic the properties of biological synapses, the energy consumption of the MHPs artificial synapses needs to be further reduced. In the artificial synapses, synaptic functions are generally caused by ion migration, which can be facilely controlled by 2D MHPs crystal structures. Synaptic functions can be achieved by electrical or photonic stimulus. The investigation on 2D MHPs artificial synapses is just at the infancy stage. The recent research progresses are discussed below, and the performance parameters of artificial synaptic devices are summarized in **Table** **2**.

**Table 2:** Summary of performance of artificial synaptic devices based on 2D halide perovskites.

Structure	stimulation type	required pulse for synaptic event	energy consumption per synaptic event [fJ]	References
ITO/BCCP/(PEA)_2_MA_n‐1_Pb_n_Br_3n+1_/Al	electronic	0.02 V, 100 ms	≈0.7	[41]
ITO/PEDOT:PSS/FA‐Bi‐I (2D+0D) mixed phase/Al	electronic	0.02 V, 300 µs	0.58 (7853 µm^2^) 0.061 (1963 µm^2^)	[78]
Au/(PEA)_2_PbBr_4_/Graphene	electronic	3 V, 10 ms	400	[70]
FTO/ZnO/BA_2_PbBr_4_/NiO	electronic	1.3 V, 100 µs	–	[67]
rGO/PEDOT:PSS/(PEA)_2_SnI_4_	photonic	470 nm, 10 ms	–	[79]
Si/SiO_2_/(PEA)_2_SnI_4_	photonic	470 nm, 20 ms	–	[96]
Si/SiO_2_/BA_2_PbBr_4_/IZTO	photonic	365 nm, 50 ms	–	[98]

### 5.1 Electrical Synapse

**Figure 9:**
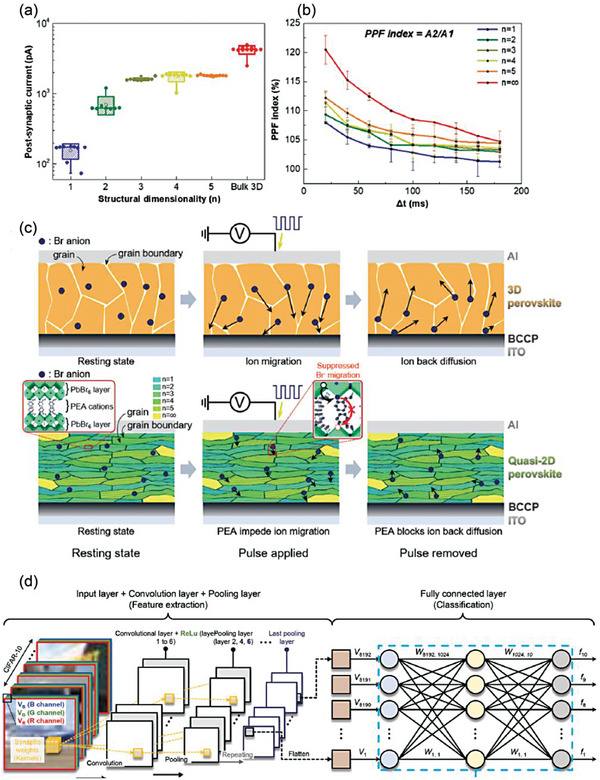
a) EPSC of MHPs artificial synapses with different dimensionality. b) PPF index (A2/A1) with time interval from 20 to 180 ms. c) Schematic mechanism of ion migration in artificial synapses. Top:3D, Bottom:quasi‐2D. Reprinted with permission.^[^ ^41^ ^]^ Copyright 2019, Wiley‐VCH. d) Memristor device consist of CIFAR‐10 dataset and a convolutional neural network. Reprinted with permission.^[^ ^78^ ^]^ Copyright 2022, Wiley‐VCH.

As discussed above, dimensionality plays an important role in memory characteristics. Meanwhile, it is also very important in artificial synapses. Kim et al. adjusted the ratio of PEA and MA cations to prepare 2D, quasi‐2D, and 3D MHP films with the formula of (PEA)_2_MA*_n_*_‐1_Pb*_n_* Br_3_ *_n_*_+1_.^[^ ^41^ ^]^ They investigated the dimensionality dependent plasticity in MHPs artificial synapses with the configuration of ITO/buffer‐capped conducting polymer (BCCP)/(PEA)_2_MA*_n_*_‐1_Pb*_n_* Br_3_ *_n_*_+1_/Al, where BCCP consisted of PEDOT:PSS with a perfluorinated ionomer (PFI), tetrafluoroethylene‐perfluoro‐3,6‐dioxa‐4‐methyl‐7‐octenesulfonic acid copolymer (PEDOT:PSS:PFI = 1:2.5:11.2 (w:w:w)). The presynaptic spike is applied on the top Al electrode and the postsynaptic current is read on the bottom ITO electrode. The current is suddenly increased and then gradually decays overtime. When paired‐pulses is applied within a short interval Δ*t*, the device shows paired pulse facilitation (PPF). This PPF property increases as Δ*t* decreases from 180 to 20 ms due to the back‐diffusion of Br^−^ decreases with strengthened learning and memory (**Figure** **9**a,b). Excitatory postsynaptic current (EPSC) increases as the spike frequency increases from 2.8 to 25 Hz. Additionally, spike‐duration dependent plasticity (SDDP), spike‐number dependent plasticity (SNDP), and spike‐voltage dependent plasticity (SVDP) are also demonstrated in this artificial synapse.

Generally, human memory is classified by sensation, short‐term memory, and long‐term memory.^[^ ^95^ ^]^ By applying 30 repeated stimuli, switching from short‐term potentiation (STP) to long‐term potentiation (LTP) presented. The memory decay curve, similar to the “forgetting curve”, can be fitted by *γ* = *bt* ^−^ *^m^*, where *γ* is the synaptic weight, *b* is the fit constant for scaling, *t* is the time, and *m* is the power function rate. The duration of memory elevates with decreasing *m*. The *m* of quasi‐2D films with *n *= 3, 4, 5 were 0.4092, 0.4953, and 0.5039, respectively, which are smaller than that of 2D and 3D counterparts. Therefore, the quasi‐2D film shows long retention. The ion migration is affected by the dimensionality. In 3D film, ions are freely to migrate around grain boundaries defects. However, the PEA in quasi‐2D inhibits the halide ions migration and increases the activation energy (*E*_A_) of Br^−^ migration, as depicted in Figure 9c. With increasing PEA amount, the Br^−^ migration is greatly suppressed. In quasi‐2D perovskites with *n* = 3–5, the Br^−^ ion migration is easier than that of low‐dimensional quasi‐2D (*n* = 1, 2) as the voltage is applied. When removing the voltage, the PEA impedes the ion reverse flow in quasi‐2D perovskites.

The relationship of energy consumption‐device dimension shows a linear dependency. In the 2D perovskite synapse, the calculated energy consumption is as low as 18 fJ per synaptic event for a device of 250 µm × 250 µm under ‐1 V. This energy consumption is comparable to that of biological synapses. Under the −0.02 V voltage pulse to, the energy consumption can be lowered to 0.7 fJ, which would enable the development of neuromorphic electronics.

Yang et al. reported a mixed‐dimensional MHPs consisting of 2D FABi_3_I_10_ and 0D FA_3_Bi_2_I_9_ as a switching active layer of artificial synapse with low energy consumption.^[^ ^78^ ^]^ Density functional theory calculation verifies the type‐I band alignment in the mixed phase. The defect density is estimated to be 3.46 × 1015 cm^−3^. The memristor devices based on mixed phase have outstanding potentiation and depression characteristics. The energy consumption is as low as 0.585 fJ with the cell area of 7856 µm^2^. Downsizing the area to 1963 µm^2^ the energy consumption lowers to 61.08 aJ. The repetitive voltage pulses can enhance iodide vacancies moved to the interface and shows SNDP. More importantly, they performed a convolutional neural network simulation. It combines the simulation with the Canadian Institute for Advanced Research‐10 (CIFAR‐10) dataset and the modified National Institute of Standards and Technology (MNIST) dataset using the “NeuroSim+ MLP” simulator. The feature extraction part is composed of the input layer, convolution layers (at from the 1st to 6th layers) and pooling layers (at the 2nd, 4th, and 6th layers) and the fully connected layers (at the 7th and 8th layers) made the classification part. The CIFAR‐10 dataset with 50 000 training data and 10 000 inference data is applied to the input layer (Figure 9d). After completing the feature extraction by repeating the process from the 1st layer to the 3rd layer once more, the pooled feature maps containing the input image are extracted from the last pooling layer (the 6th layer) and these feature signals are flattened into a 1 × 8912 array for the classification process. In addition, the recognition rate of the network using the mixed phase synapses is ≈87%, higher than their counterparts. In the mixed phase‐based synapse, the high dynamic range contributes to this high recognition rate, so as to low asymmetricity.

### 5.2 Optical Synapse

**Figure 10:**
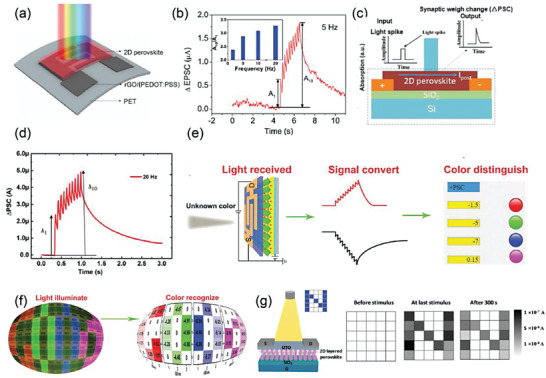
a) Schematic of the device structure based on (PEA)_2_SnI_4_. b) ΔEPSC under 10 light pulses. The inset shows the frequency‐dependent EPSC gain (A10/A1). Reprinted with permission.^[^ ^79^ ^]^ Copyright 2018, The Royal Society of Chemistry. c) Schematic illustration of the (PEA)_2_SnI_4_ artificial synaptic devices. The insets show the input light spikes and the output PSC. d) The ΔPSC triggered by a train of 10 light spikes (20 ms, 20 Hz). Reprinted with permission.^[^ ^96^ ^]^ Copyright 2019, Wiley‐VCH. e) Simulation process of the synaptic transistor's recognition. f) 12 × 5 transistor arrays of LED light. Input images in the letter “G,” “o,” “o,” and “d” consisting of four 3 × 5 pixels encoded by red, green, blue, and NIR light, respectively. Reprinted with permission.^[^ ^97^ ^]^ Copyright 2021, Wiley‐VCH. g) Current response before stimulus, at the last stimulus, and 300 s after last stimulus by inputting letter of “N”. Reprinted with permission.^[^ ^98^ ^]^ Copyright 2021, The Royal Society of Chemistry.

2D MHP can also be applied in high‐performance photodetectors and light‐stimulated synaptic device. Qian et al. presented a photoconductor based on (PEA)_2_SnI_4_ on rGO (reduced graphene oxide)/PEDOT:PSS (**Figure** **10**a).^[^ ^79^ ^]^ By the introduction of SnF_2_, Sn vacancies are effectively reduced. The flexible photoconductors perform a photoresponsivity of 16 A W^−1^ and a detectivity of 1.92 × 10^11^ J under 470 nm illumination. Moreover, light pulse can be transmitted to the device by generating an EPSC. ΔEPSC increases from 0.8 to 7.0 µA when the light pulse duration time increases from 1 to 200 ms. When a series of same duration light pulses with different irradiance are applied on the device, ΔEPSC decreases from 3.7 to 1.7 µA with decreasing irradiance. The synapse device shows typical STP behavior. When the device is stimulated by 10 light pulses with 200 ms intervals, the peak value of EPSC actuated by one pulse is constantly increasing. The EPSC decays to the initial current when light is removed. The EPSC gain (A10/A1) increases with the light frequency (Figure 10b). The Sn vacancies are ascribed to the synaptic behavior.^[^ ^99^ ^]^

Sun et al. studied the photoelectric synaptic plasticity of (PEA)_2_SnI_4_ under light stimuli, as shown in Figure 10c.^[^ ^96^ ^]^ The device shows typical PPF synaptic plasticity. By 470 nm light stimuli, the 2D perovskite film conductivity will be changed to behave as PSC. Under a train of 10 light spikes with 20 Hz, 20 ms, the ΔPSC is improved significantly, which shows a short‐term learning and memory effect (Figure 10d). By the fixed light irradiance 11.6 µW cm^−2^ and adjusting the spike durations, a larger ΔPSC and a longer memory retention time occurs with a longer spike duration. It also shows STP characteristic by removing the light stimulus. STP can change to LTP with varying frequencies of the light spikes under the irradiance of 11.6 µW cm^−2^. This phenomenon is vital for human brain memory. They attributed Sn vacancies to be responsible for the deeper traps. The artificial synapses behavior can be affected by the density of Sn vacancies in the (PEA)_2_SnI_4_ film.

In information sensing artificial intelligence vision systems (AIVSs), to enlarge the absorption spectrum from visible to near‐infrared (NIR), Huang et al. developed PEA_2_SnI_4_/Y6 heterojunction synapse phototransistor.^[^ ^97^ ^]^ The perovskite‐organic heterojunction structure shows high photo response of 104 A/W to visible light, and 200 A W^−1^ to NIR light. The stimuli of a single red light pulse generates an EPSC. In darkness, the hole–electron pairs in the PEA_2_SnI_4_/PVP interface will discharge slowly, exhibiting EPSC decay behavior. After two successive synaptic stimulations, the device shows PPF. Under the application of 50 and 100 light spikes, the ΔPSC value was shown to increase with increasing light spike. This behavior is very similar to the human learning behavior, which can be used to model the artificial intelligence vision systems. The synaptic phototransistors can convert the received light signal into EPSC/inhibitory postsynaptic current (IPSC). The light color can be distinguished by the postsynaptic current value. They fabricated a flexible 12 × 5 arrays and illuminated ten light spikes by four different color light‐emitting diodes to encode four letters of “G”, “o”, “o”, and “d” (Figure 10e). The red, green, and blue light spikes illumination generates IPSC currents of different magnitudes. Otherwise, NIR light spikes induce EPSC currents. The different current magnitudes mean the different four letters showing on each pixel. Therefore, G (red), o (green), o (blue), and d (NIR) can be distinguished, which can be seen in Figure 10f. This work explores a wonderful application of PEA_2_SnI_4_ in simulating the retinal function of the human eye.

Optoelectronic synaptic device with long‐term visual memory was also be realized by Park et al. with BA_2_PbBr_4_/indium zinc tin oxide (IZTO) heterojunction.^[^ ^98^ ^]^ The BA_2_PbBr_4_ layer was fabricated by vapor deposition, which is different from the popular solution process. To emulate the PPF property, two consecutive optical pulses were conducted to the device with Δ*t* = 400 ms. The PPF index is 55% and 21% for Δ*t* = 100 ms and Δ*t* = 2 s, respectively. The photonic synapses can be applied in practical artificial neural networks (ANNs). They conducted a multilayer perceptron neural network with 400 input neurons, 100 hidden neurons, and 10 output neurons with the MNIST data set. The recognition accuracy is high to 80% for controlled‐pulse condition. The human visual memory can be simulated by the conductance change under illumination. A 5 × 5 pixel array to show the letter “N” was fabricated. At first, all devices were in the OFF state. After the simulation of optical pulses, the letter “N” was recognized. The memory can maintain for 300 s, as shown in Figure 10g. This work explores the development of human visual memory in 2D MHPs.

## 6 Summary and Outlook

There are 3D MHP, 2D MHP, and all‐inorganic perovskite investigated in RSMs. 3D MHP is the most typical perovskite among the three types. As far as the memory behavior is concerned, the ON/OFF ratios of 3D MHPs reported show higher than 2D MHPs according to the recent studies. However, 3D MHP is sensitive to ambient conditions. The device stability and reliability are reported few. The other two types of perovskites are more stable than 3D MHP. All‐inorganic perovskite with representative cesium could retain the structural and thermal stability even above 100 °C.^[^ ^100^ ^]^ It shows outstanding merits in the application of high temperature conditions. 2D MHPs, an important type of perovskites with reduced dimensionality, have shown flourishing applications in miniaturized, high‐density, and RS memories, which mainly because of their excellent physical and electrical properties. This article summarizes the recent advances of 2D MHPs RSMs and artificial synapses.

Investigations on 2D MHPs have revealed that their distinct properties, grain sizes, interface engineering, and interlayer spacing significantly alter the material properties, which are important for memory applications. Concerning the demands for next‐generation nonvolatile memory devices, the RSM should have ON/OFF ratio greater than 10, endurance larger than 10^3^, and retention time exceeding 10 years.^[^ ^2^ ^]^ According to the recent studies, only the ON/OFF ratio of 2D MHPs RSMs could meet the requirement. The endurance and retention characteristic still need great effort to improve. Moving forward, it is necessary to address major issues and challenges.

The crystal structure of the 2D MHPs can present parallel to the substrate with little *n* in the 2D MHPs molecular formular. By increasing *n*, it will show perpendicular to the substrate. The parallel crystal growth direction is disadvantageous for charge transport compared with the perpendicular growth direction. While the orientation on the memory features has not been reported yet. The charge transport direction may expand the research on 2D MHPs RSM devices. A vital issue for the advancement of high performance and high stability optoelectronic applications is to understand how the organic spacer cations affect the properties of 2D perovskites. The quasi‐2D MHPs are prepared with stoichiometric amounts of precursor, containing different *n* values with smaller bandgap. They typically consist of multiple quantum wells which distribute randomly. The fabrication of phase‐pure quantum wells may be a key scientific challenge in 2D HPs memories.

In order to build‐up the RSM devices, the manufacturing process of the MHPs films is of great importance for the formation of high‐quality perovskites. Generally, spin coating is a simple and most popular method used in laboratory, which offers easy control of the film thickness. However, antisolvents dipped into the perovskite precursor solution are usually required in spin coating as well as an inert atmosphere. The high‐quality perovskite films suffer from the very narrow time window for properly dipping the antisolvent, rendering in serious reproducibility problems.^[^ ^101^ ^]^ Herein, developing one‐step facile film preparation methods with environment‐friendly solvents in ambient atmosphere for fabricating uniform perovskite films is a challenging research subject. Recently, Chen et al. have investigated the ionic liquid as the precursor in fabricating 3D and 2D perovskite films by one‐step spin coating without antisolvents.^[^ ^102^, ^103^, ^104^ ^]^ Phase‐pure quantum well films will be formed by introducing a molten salt spacer *n*‐butylamine acetate. These findings imply that the innovative fabrication method would contribute to the 2D MHPs RS memories. Moreover, inkjet printing, screen‐printing, as well as blade coating can be explored for fabricating solution‐processable large‐scale 2D MHPs film.^[^ ^105^, ^106^, ^107^ ^]^

The interface engineering plays an important role in memory behavior. Much efforts should be devoted except for the traditional interface layers such as ZnO, PMMA. Electrode materials significantly affect RS characteristics.^[^ ^108^ ^]^ With different electrodes, RS memories may behave as unipolar, bipolar, WORM, or multilevel properties. However, the underlying dependence has not been thoroughly developed in blooming 2D MHPs memories. Besides the most popular electrodes (Au, Ag, Al), other uncommon electrode materials like Pt, Cu, W, Ni, and metal oxides should be explored. To commercialize 2D MHPs memristors, it is necessary to ensure film uniformity at the wafer‐scale level. Additionally, mechanical reliability of 2D MHP films is vital for flexible electronics. The processing technology described above can optimize film uniformity and flexible substrates.

The majority of 2D MHPs being reported contain lead. The natural toxic nature of lead prevents them from commercial application. In the current research, few lead‐free 2D MHPs are investigated for memristors. Tin is the most common element proposed to replace lead. But tin is easily oxidized due to the higher energy level in the 5s orbit. So, Sn‐based 2D MHPs are susceptible to degradation by exposure to air and water. Compared with Sn ion, Bi^3+^ shows more stable. Interestingly, a double perovskite can be constructed by replacing two lead sites with a monovalent cation and a trivalent ion. It generally shows perfect stability as well as higher switching ratio. In terms of the tunability of the lattice and composition of MHPs, developing new lead‐free 2D MHPs is rather challenging.

For the artificial synapses application of 2D MHPs, an important challenge is how to lower the extremely high resistance. Charge doping to adjust the conductivity is a popular way. Besides, concerning the film thickness factor, the resistance will decrease with decreasing film thickness. However, under the widely used solution fabrication method, the thickness is easily to be restricted. Therefore, novel film preparation techniques, such as vacuum evaporation may play vital role in 2D MHPs film resistance controlling.

In particular, 2D MHPs show good performance in memories and artificial synapses, which means they are promising candidates for the cutting‐edge information era. Until now, the research of 2D MHPs memory and synaptic devices are still in their infancy. It is hoped that the above discussion and outlook can deepen our understanding of 2D MHPs devices. We aim to provide an overview and an extraordinary development direction for future advancement of 2D MHPs RSMs.

## Conflict of Interest

The authors declare no conflict of interest.
